# A study on Ovine pneumonic pasteurellosis: Isolation and Identification of Pasteurellae and their antibiogram susceptibility pattern in Haramaya District, Eastern Hararghe, Ethiopia

**DOI:** 10.1186/1746-6148-9-239

**Published:** 2013-12-01

**Authors:** Haimanot D Marru, Takele T Anijajo, Adem A Hassen

**Affiliations:** 1 College of Health Sciences, School of Veterinary Medicine, Wollega University, P. O. Box 395, Nekemte, Ethiopia; 2 School of Biomedical and Laboratory Sciences, College of Medicine and Health Sciences, University of Gondar, P.O. Box 196, Gondar, Ethiopia; 3 Faculity of Veterinary Medicine, Haramaya University, P. O. Box 138, Dire Dawa, Ethiopia

**Keywords:** Antimicrobial, Haramaya district, Hararghe, Bacterial isolation and identification, Pneumonic pasteurellossis

## Abstract

**Background:**

Sheep constitute the second major component of livestock in Ethiopia. However, efficient utilization of this potential resource is hampered by combination of health problems, poor management and feed shortage. Haramaya district is one of the remote settings in Ethiopia where information about the livestock disease is not well documented. Hence this study was conducted to determine the causative agents and their antimicrobial susceptibility pattern of bacterial *Pasteurella* isolates among pneumonic ovine in Haramaya district, Eastern Hararghe, Ethiopia.

**Results:**

Out of 256 samples examined, *Pasterurella* was isolated in 64 (25%), of which 38 (59.4%) were from lungs and 26 (40.6%) were from nasal cavities. 87.5% of the isolates were *Mannheimia haemolytica* and 12.5% were *Pasteurella multocida*. All of the isolates from the lungs were *Mannheimia haemolytica* whereas 69% of the isolates from nasals cavities were *Mannheimia haemolytica*. Age and body temperature were significantly associated with *Pasteurella* isolates from clinic (P < 0.05). Despite diverse in the site of origins, the isolates exhibited uniformity in sensitivity to a majority of the antibacterial agents. The most effective drug was Cholramphenicol (100%) followed by Sulfamethoxazole (89.1%) and Tetracycline (84.4%). Both species were completely resistant to Gentamycin and Vancomycin.

**Conclusion:**

*Mannheimia haemolytica* is the most common cause of ovine pneumonic pasteurellosis in the study area. The isolates were susceptible to limited antimicrobial agents. Therefore, the antimicrobial susceptibility test should be conducted before treatment, except for critical cases.

## Background

Pneumonia is extremely common in sheep and can be responsible for enormous financial losses worldwide. The condition usually appears when sheep are exposed to combinations of predisposing factors such as adverse physical condition, physiological stress, bacterial and viral infections. As the exact nature of these combinations is unknown, much remain to be understood about why the disease occurs in the way it does [1]. Sub-Saharan Africa is endowed with a large population of sheep. The population of sheep is estimated at 132.5 million head [2]. However, animal productivity per head is low.

Ethiopia lies within the tropical latitude of Africa and has an extremely diverse topography, a wide range of climatic features and a multitude of agro-ecological zone which makes the country suitable for different agricultural production system. This in turn has contributed to the existence of a large diversity of farm animal genetic resource in the country [3]. Sheep constitute the second major component of livestock in Ethiopia. Despite the large livestock population of Ethiopia the economic benefits remain marginal due to prevailing diseases, poor nutrition, poor animal production systems, reproductive inefficiency management constraint and general lack of Veterinary core [4].

Pneumonic pasteurellosis is one of the priority diseases that deserve control. However, control of pneumonic pasteurellosis is difficult task that requires integration of various techniques. Developed and developing countries practice various control mechanisms for primary diseases. While developing countries including Ethiopia could not apply the strategies used by developed countries because of economic reason. The most economic and feasible control method for developing nations is the use of vaccine (Fekadu and Girum*:* Study of the prevalent serotype of Ovine pasteurellosis in the high land of Eastern Amhara sub-region. Kombolcha Veterinary laboratory, unpublished*:* 2001).

In Ethiopia there are some works conducted on ovine pneumonic pasteurellosis: seroprevalence, bacteriological prevalence in pneumonic lungs and detection of most prevalent serotyping throughout the selected sites of Ethiopia. But, no work was done in Eastern Hararghe at Haramaya district on the *Pasteurella* isolation as well as the drugs or antimicrobial to which these isolates are susceptible for. Therefore, this study was focused on the isolation and identification of *Pastuerella* species that involved in ovine pneumonic pasteurellosis among apparently healthy sheep with pneumonic lungs slaughtered at Haramaya municipal abattoir and sheep with clinical manifestation presented to the Haramaya veterinary clinic during the study period. In addition, this study attempted to determine drug susceptibility pattern of these isolates.

## Methods

### Study area

The study was conducted at Haramaya district which is located in Eastern Hararghe Zone of Ethiopia, approximately 500 kms East of Addis Ababa. Its elevation is approximately 2000 m a.s.l. and the mean annual temperature and relative humidity are 18°C and 65%, respectively. An annual rainfall is approximately 900 mm, with a bimodal distribution pattern, peaking in mid April and mid August. There are four seasons, such as a short rainy season (from mid March to mid May), a short dry season (from end May to June), a long wet season (July to mid October) and a long dry season (end of October to February). Main pasture production is expected after the short rainy season, continuing until the end of the long wet season. Mixed type agriculture is the main occupation of the population of the area. According to the veterinary sector of Haramaya district agricultural office 2000 report, a total livestock population of this district was 193,334; of which 64,510 Cattle, 28,359 goats, 18,930 sheep, 15,277 donkeys, 5 mules, 530 camels and 65,723 poultry [5].

### Study design

A cross-sectional study was undertaken from November 2007- April 2008 on ovine pneumonic pasteurellosis to isolate and identify the *Pasteurella* species from specimens of nasal swab of clinically suspected pneumonic sheep from Haramaya veterinary clinic and lung swab of pneumonic lungs of apparently healthy sheep slaughtered at Haramaya municipal abattoir.

### Study animal

The animals used in this study were apparently healthy sheep slaughtered at Haramaya municipal abattoir and sheep with clinical manifestations presented to the Haramaya veterinary clinic during the study period. At the abattoir, the lungs of the slaughtered animals were visualized, palpated and inspected thoroughly and finally the pneumonic lungs were considered. According to the postmortem meat inspection principle the lungs of the slaughtered animal were visualized and palpated for haemorrhage, edema and pneumonia. Up on visualization, palpation and inspection the lungs with consolidated inflamed area, deep red and sharply demarcated lesion were considered as pneumonic lungs [6]. All sheep irrespective of age, sex, and color were examined at clinic for the evidence of pneumonic pasteurellosis. Up on clinical examination, all sheep manifesting; anorexia, coughing, dyspnea, lethargy, serous to muco-purulent ocular and nasal discharge, and fever were considered. In both cases the animals with pneumonic lung and the mentioned clinical features were considered to be study population. The sample size for each varied according to the availability of suspected cases of sheep with pneumonia in respective to study site. A total of 256 (83 from clinic and 173 from abattoir) suspected cases were collected.

### Ethical consideration

Investigators treated animals with kindness and took proper care by minimizing discomfort, distress or pain. They assumed that all procedures which would cause pain in human beings may cause pain in study animals. The required procedures were conducted by qualified and experienced persons [7]. The ethical clearance was obtained from Haramaya University ethical review board.

### Data collection

#### Nasal swab

Each animal was individually identified and restrained by an assistant and kept fixed. After disinfection of external part of the nose with 70% alcohol, a sterile cotton-tipped swab was inserted in to the nostril and rotated against the wall of the nasal cavity [8]. The swab was placed in labeled sterile test tube that contains 3 ml of tryptose Soya broth, and then kept in an ice box for transport to Haramaya University, FVM. Laboratory [8].

#### Lung swab

Following the slaughter of the apparently healthy animal, all the lungs were inspected according to the standard postmortem meat inspection procedure. Up on inspection the surface of each suspected lung was incised using sterile scalpel blade and the inner surface of the incision was sampled with sterile swab. The swab was transported to Veterinary Microbiology Laboratory of Haramaya University in the same procedure with nasal swab [9].

### Isolation and Identification of Pasteurella

The isolation and identification of *Pasteurella* were performed at the Veterinary Microbiology Laboratory of Haramaya University using techniques recommended by Hardy Diagnostics, Santa Maria, CA, USA. The isolation and identification involves the following steps: first, the pre-enriched in tryptose Soya broth specimen was incubated for 24 hrs at 37°C. After 24 hrs incubation, a loop full of the broth cultures were taken and streaked over an identified Petri-plate containing blood agar base supplemented with 7% sheep blood and immediately incubated aerobically at 37°C for 24 hours [9]. Secondly, from culture positive plates, typical colonies were subjected to gram’s staining to study staining reactions and cellular morphology under light microscope, at 100x magnification. Mixed and gram-negative bacteria were further sub cultured with due care, on both blood and MacConkey agar plates [9] for further analysis. The growth of typical colonies on both blood and MacConkey agar was characterized using blood agar for the presence of haemolysis, the type of haemolysis, the general appearance of colonies (morphology, color, shape size and consistency) and the ability to ferment lactose [10]. Thirdly, pure cultures of single colony type from both Blood and MacConkey agars were transferred onto nutrient agar-slants for a series of primary biochemical tests: catalase (Hydrogen peroxide, Fisher Chemical, UK), oxidase (TM-pphenylenediamine dihydrocholoide, Merck Co., Germany) and fermentative/oxidative (OF Basal Medium, Titan Biotech Ltd, India). Final identification of the bacteria to the species level was aided by using the secondary tests which include: metabolism of sugars such as glucose and L-arabinose; and alcohols and tests for metabolic end products such as Indole (Peptone water, Merck Co., Germany) following standard procedures [11,12]. The assay for biochemical properties of the bacterial isolates were conducted according to MacFaddin’s method [13]. For reliable identification and comparison of results, the AIPE 20 system (Biomariux France) was used. If the organism is able to produce a narrow zone of haemolysis on Blood agar and grow on McConkey agar, but unable produce indole, interpreted as *P.haemolytica*. If the organism is unable to produce haemolysis on Blood agar and cannot grow on MacConkey, but able to produce indole, interpreted as P. *multocida.*

### Antimicrobial susceptibility test

The antimicrobial susceptibility test on the isolates was performed according to the National Committee for Clinical Laboratory Standards (NCCLS, 1990) [14] method using Kibry-Bauer disk diffusion test on Muller-Hinton agar (Oxoid CM0337 Basingstoke, England). *Escherichia coli* ATCC 25922 was used as a quality control organism for the antimicrobial susceptibility test [15]. The isolates were tested for the following antibiotics; Chloramphenicol (CAF) 30Ng, Tetracycline (TTC) 30Ng, Penicillin-G (P) 10 unit, Ampicilin (Am) 10Ng, Sulfamethoxazole(SxT) 5Ng, Streptomycin(S) 10Ng, Vancomycin (VA) 30Ng and Gentamicin (CN) 30Ng based on the procedure recommended by Carter [16] and Quinn *et al.*[17]. The zone of inhibition was interpreted based on the Performance Standards for Antimicrobial Susceptibility Testing; Sixteenth Informational Supplement [18] as detailed in Table 1.

**Table 1:** Zone interpretive chart for antimicrobials (inhibition zone diameter in mm)

**Antimicrobial agent**	**Disc potency**	**Resistant**	**Intermediate**	**Susceptible**
	**(μg)**	**(≤)**		**(≥)**
Tetracycline	30	14	15-18	19
Streptomycin	10	11	12-14	15
Chloramphenicol	30	12	13-17	18
Vancomycin	30	14	15-16	17
Penicillin-G	10u	28	-	29
Ampicillin	10	13	14-16	17
Sulphamethoxozole	25	10	11-15	16
Gentamicine	10	12	13-14	15

Source: Clinical and Laboratory Standards Institute (CLSI) [18].

### Data entry and analysis

Data collected from both the clinic and abattoir were recorded in the format developed for this purpose and later on entered into the Microsoft excel 2000 program and analyzed using STATA 7.0 software. Association of host risk factors with swab culture positives was calculated. A statistically significant association between variables was considered to exist if the computed p-valve was less than 0.05.

## Results

### Bacterial isolation

From 256 samples (83 nasal swabs and 173 lung swab) collected and cultured, *Pasteurella* was isolated successfully in 64 (25%) sheep. Out of 64, 26 (40.6%) were from nasal cavities and 38 (59.4%) from lungs. Eighty seven point five percent of the isolate was *M. haemolytica* and 12.5% was *P.multocida* and of the lung samples which were culture positive, 100% was *M. haemolytica.* In nasal cavities, of the samples which were culture positive, 69% of the isolate was *M. haemolytica* and 31% of the isolate was *P.multocida* (Table 2). On the basis of these results *M. haemolytica* was the most common cause of pneumonic pasteurellosis in sheep at Haramaya district.

**Table 2:** ***Pasturella*** **Isolates from nasal cavity and lung samples of Ovine at clinic and abattoir at Haramaya district in Eastern Hararghe, Ethiopia**

***Site***	***Species***		***Total***	**Percentage of total isolates**	**X2 (P-value)**
	***M.haemolytica***	***P.multocida***			
	**Positive**	**Positive**			
Nasal cavity					
Positive	18(69%)	8(31%)	26(31.3%)	26(40.6%)	
Negative			57(68.7%)		
Total			83(100%)		2.6208 (0.105)
Lung sample					
Positive	38(100%)	-	38(21.96)	38(59.4%)	
Negative			135(78.04%)		
Total			173(100%)		
**Total**	56(87.5%)	8(12.5%)	256(100%)	64(25%)	

*M. haemolytica* was isolated in both clinical and abattoir, but *P.multocida* was exclusive to clinical cases. The magnitude of *M. haemolytica* was almost equal in both clinical (21.69%) and abattoir (21.96%) cases; however, the overall magnitude of the bacteriological confirmed cases is higher in clinical cases (31.3%) than abattoir cases (21.96%). The result indicated that, there is positive association between body temperatures of the animals at clinic and bacteriological confirmed cases of pneumonic pasteurellosis (P = 0.007), but age has negatively associated (P = 0.028) particularly with *P.multocida* (P = 0.004) infection and sex has no any association with both species (P > 0.05) (Table 3). No associations were observed between the risk factors and pneumonic pasteurellosis at abattoir (P > 0.05) (Table 4).

**Table 3:** Association of culture positive results with the risk factors (age, sex and body temperature) from clinic cases at Haramaya district in Eastern Hararghe, Ethiopia

***Age***	***Young***	***Adult***	***Total***		***Chi***^***2***^	**P-value**	**Overall**
*M. haemolytica*							***Chi***^***2***^^***=***^^***4.8501 P = 0.028***^
Negative	17	48	65		2.2405	0.134	
Positive	8	10	18				
Total	25	58	83				
*P.multocida*							
Negative	19	56	75		8.472	0.004	
Positive	6	2	8				
Total	25	58	83				
**Sex**	**Male**	**Female**	**Total**		**Chi**^**2**^	**P-value**	Chi2 = 2.6186 Pr = 0.106
*M.haemolytica*							
Negative	40	25	65		2.9443	0.086	
Positive	7	11	18				
Total	47	36	83				
*P.multocida*							
Negative	44	31	75		1.3187	0.251	
Positive	3	5	8				
Total	47	36	83				
**Temprature**	**38.5-39.5o**^**C**^	**39.6-40o**^**C**^	**>40o**^**C**^	**Total**			***Chi***^***2***^ ***=*** 10.0614 p = 0.007
Negative	21	23	13	57			
Positive	4	7	15	26			
**Total**	25	30	28	83			

**Table 4:** Association of culture positive results from abattoir with risk factors (age and sex) in Abattoir cases at Haramaya District in Eastern Hararghe, Ethiopia

***Age***	***Young***	***Adult***	***Total***	***Chi***^***2***^	**P-value**
*M.haemolytica*					
Negative	12	123	135	2.7560	0.097
Positive	7	31	38		
Total	19	154	173		
*P.multocida*					
Negative	19	154	173	-	-
Positive	-	-	-		
Total	19	154	173		
**Sex**	**Male**	**Female**	**Total**	**Chi**^**2**^	**P-value**
*M.haemolytica*					
Negative	118	17	135	1.1348	0.567
Positive	31	7	38		
Total	149	24	173		
*P.multocida*					
Negative	149	24	173	-	-
Positive	-	-	-		
**Total**	149	24	173		

### Antimicrobial susceptibility pattern

Sixty four isolates of *Pasturella* isolates from clinic and abattoir were subjected to a panel of eight antimicrobials. The antimicrobial susceptibility pattern of the isolates indicated that all isolates were 100%, 100%, 90.6%, and 87.5% resistant to gentamycin, vancomycin, streptomycin and penicillin-G respectively. On the other hand, the isolates were 100%, 89.1%, 84.4% and 53.1% sensitive to chloramphenicol, sulfamethoxazole, tetracycline and ampicilin respectively (Table 5). Despite diverse in the site of origins, the isolates exhibited uniformity in sensitivity to a majority of the antibacterial agents. *M. haemolytica* showed 100% resistance to gentamycin and vancomycin while they were 100% sensitive to chloramphenicol followed by 89.3% and 83.9% to sulfamethoxazole and tetracycline consequently. Similarly *P.multocida* showed 100% sensitivity to chloramphenicol followed by sulfamethoxazole and tetracycline. However, *P. multocida* is more susceptible to sulfametrioxazole and tetracycline, but not statically significant (P > 0.05).

**Table 5:** **Antimicrobial susceptibility pattern of *Pasturella *** **Isolates from nasal cavity and lung samples of Ovine at clinic and abattoir at Haramaya district in Eastern Hararghe, Ethiopia**

**Antimicrobials tested**	**Species of bacteria**		**Total**
	***M.haemolytica***	***P.multocida***	
Ampicllin			
Sensitive	30(53.6%)	4(50%)	34(53.1%)
Resistance	26(46.4%)	4(50%)	30(46.8%)
Chloramphenicol			
Sensitive	56(100%)	8(100%)	64(100%)
Resistance	-	-	-
Gentamycin			
**S**ensitive	0(0%)	-	-
Resistance	56(100)	8(100%)	64(100%)
Tetracycline			
Sensitive	47(83.9%)	7(87.5%)	54(84.4%)
Resistance	9(16.1%)	1(12.5%)	10(15.6%)
Penicillin-G			
Sensitive	6(10.7%)	2(25%)	8(12.5%)
Resistance	50(89.3%)	6(75%)	56(87.5%)
Streptomycin			
Sensitive	5(8.9%)	1(12.5%)	6(9.4%)
Resistance	51(91.1%)	7(87.5%)	58(90.6%)
Sulfamethoxazole			
Sensitive	50(89.3%)	7(87.5%)	57(89.1%)
Resistance	6(10.7%)	1(12.5%)	7(10.9%)
Vancomycin			
Sensitive	-	-	-
Resistance	56(100%)	8(100)	64(100%)

## Discussion

In the present study, *Pasteurella* species were isolated in 25% of the sheep, of which 59.4% and 40.6% were contributed from abattoir and clinic respectively. *M. haemolytica* was accounted for 87.5%. The prevalence of *M. haemolytica* was almost similar both at abattoir (21.96%) and clinic (21.69%). *P. multiocida* was discovered only from nasal swabs. Pneumonic pasteurellesis was positively associated with body temperature and negatively associated with the age of suspected ovine particularly *P. multiocida*. Sex has no any association.

In the present study, *M. haemolytica* was isolated from 21.96% of lung samples examined. This result is greater than the findings of Eshetu [19] and Nurhusien [20], which were reported as 13% and 8.7% respectively, but lower, than the findings of Mohammed [21] and Aschalew [22] that were reported as 40.8% and 56% respectively in pneumonic lungs. In nasal swab *M. haemolytica* was discovered with the rate of 21.7%, which is similar with that of the lung swab. Nonetheless, the present result indicates high prevalence of the isolate than the work done by Eshetu [19] who reported 13% in the same area before sixteen years ago. This difference might be due to the type of sample taken from purely pneumonic lung in the present study. The other possible explanation may be improvement of the health facilities within the last ~ two decades.

Comparing the two *Pasteurella* spp, *M. haemolytica* constituted 87. 5% of the total indicated that, *M. haemolytica* was the major causative agent involved in ovine pneumonic pasteurellosis. This is consistent with previous reports of Aschalew [22], Eshetu [19], Mohammed [21], and Tesfaye [23]. *M.haemolytica*, which is a normal flora of the upper respiratory tract, may play a secondary role after the primary initiating agent suppressed the host defense mechanism, and favors the multiplication of *Pasteurella* species leading to bronchopneumonia in purely pneumonic animal [24]. Although the percentage isolation was relatively low (12.5%), the possible role of *P. multocida* in the a etiology and pathogenesis of ovine pneumonia should not be under estimated.

In our study *Paseurella* species were isolated in 31.3% of nasal swabs. Although they may be found occasionally as a normal inhabitant of the respiratory system, experimental evidence has shown that under certain conditions associated with debilitation, nutrition and climatic factors, these organisms may singly or in concert with other organisms flare up to cause severe infections with high morbidity and mortality. In this particular study the recruitment of animals was based on their definitive clinical sign and symptom to pneumonia and the identification of *Pateurella* species among these animals makes more soundable and interesting. The magnitude of *M. haemolytica* was almost equal in nasal swabs (21.69%) and lung swabs (21.96%). *P. multiocida* was not isolated in the lung samples in the present study, but it was discovered in nasal swab at the rate of 9.6%. This result, however, supports the idea of previous studies [21,25] that reported *P. multocida* from respiratory tract of sheep and goats with very lower isolation rates from lung.

In the current study a significant association between pneumonic pasteurellesis and body temperature of suspected ovine irrespective of *Pasteurella* species was observed. This result coincides with the findings of (Fekadu and Girum: Study of the prevalent serotype of Ovine pasteurellosis in the high land of Eastern Amhara sub-region. Kombolcha Veterinary laboratory*,* unpublished:2001) that explained, pneumonic pasteurellosis is characterized by fever, driving or pushing of the body as result of forced respiration, dry coughing and muco-purulent nasal discharges. Similarly, the disease is significantly associated with the age of sheep. This result is also in agreement with findings of Gilmour and Gilmour [26], that elucidates pneumonic pasteurellosis occur in all ages of sheep and goats, with the most susceptible in lambs and kids during first life, and dams at lambing.

It is important to monitor the antimicrobial susceptibility of *Pasteurella* species to determine resistance development. Increase in resistance against antibiotics in both *P. multocida* and *M. haemolytica* isolates have been reported [27,28]. In our study, according to the antimicrobial susceptibility test results, chloramphenicol (100%), sulfamethoxazole (89.1%) and tetracycline (84.4%) were the most effective drugs, whereas ampicilin (53.1%) was the only intermediate drug. Penicillin-G (12.5%) and streptomycin (9.4%) were inefficient drugs; gentamycin and vancomycin were totally inactive against both isolates. This was in line with the literature which stated as chloramphenicol is highly effective and well-tolerated broad spectrum antibiotic to many genera of gram-positive and gram-negative bacteria [24]. Susceptibility to sulfamethoxazole over 80% was very close to the rate reported previously [28]. However, this result contradicts the findings of Aschalew [22] who reported that tetracycline as ineffective drug of choice. This difference strengthens the recommendation of Kaan *et al.*[29] which stated that “Antibiotic susceptibility profiles of *P. multocida* and *M. haemolytica* help veterinarians to choose appropriate antibiotic against bovine respiratory disease; however, antibiotic susceptibility studies should be renewed periodically”. In addition, pre-existing resistances in which the cellular mechanisms required for antimicrobial susceptibility are absent from the bacterial cell or acquired genetically because of chromosomal mutation and accusation of transferable genetic material. Treatment with a specific antimicrobial agent selects those micro-organisms that have pre-existing or acquired resistance [9].

One of the interesting findings of our study was the demonstration of the highest resistance of *Pasteurella* isolates against gentamycin (100%) and vancomycin (100%). In contrast, Esra *et al.*[30], reported that gentamycin (95.0%) as the most effective antibiotic against *M. haemolytica* isolates. Similarly Post *et al.*[31], reported 90.0% of *M. haemolytica* isolates revealed from cattle with bovine respiratory disease complex were markedly susceptible to gentamycin. This might be due to difference in the strain of the isolate that may cause pasteurellosis in different species of animals or due to the existence of host factors that may affect the action of drug in sheep. However this study strengthens the statement vancomycin is active against most gram-positive bacteria, but is not effective against gram-negative cells because of their large size and poor penetrability [28]. In this study *P. multocida* showed resistance to penicillin-G, this result is in contrary to literature [32] which indicates most strains of *P. multocida* are susceptible to penicillin-G.

Limitation of the study: this study was conducted in short period of time; hence we did not saw the seasonal variation of the isolates. In addition, even though identification of serotypes is so important, in our study it was not conducted due to lack of facilities in our laboratory. A part from these limitations our study has the following strengths: the sample was collected from both abattoir and clinic which shows the reliability of the data, the isolates were obtained from pneumonic ovine that made us compare the clinical case with ethological agents, all laboratory works were followed standard procedures and quality control was exhibited in each step of the work.

## Conclusions

In conclusion, Pneumonic Pasteurellosis was the major disease of sheep in the area and *M. haemolytica* is the most common cause. Being young animal was a risk factor for the disease. Both species were susceptible to limited antimicrobial agents. Chloramphenicol, sulfamethoxazole and tetracycline were effective drugs whereas gentamycin and vancomycin were totally inactive against the isolates. Measures such as, improving management practices by providing optimal sanitation and air quality in housing, minimizing transportation stress, providing good quality hay and water, and supplement as appropriate should be taken into account to reduce the prevalence. In this line bacterial isolation and antibiotic susceptibility test should be conducted before treating with antibiotics except for critical ones. Moreover, further serotypoing and molecular techniques are needed to identify the isolate to the strain level.

## Competing interests

All authors have declared that no competing interests exist.

## Authors’ contributions

HD: Conception of the research idea, designing and data collection, data analysis and interpretation, and manuscript reviewing. TT: Data collection, interpretation of the results, and drafting the manuscript. (AA): Data collection, interpretation of the results and drafting the manuscript with TT. All authors read and approved the final manuscript.
